# Fetal heart quantification technique improves the prenatal prediction of coarctation of the aorta: A retrospective analysis

**DOI:** 10.17305/bb.2024.10988

**Published:** 2024-08-18

**Authors:** Xiaoxi Lu, Bowen Zhao, Mei Pan, Lijian Huang, Xiaomin Zhang, Xiaohui Peng, Ran Chen, Xiangdong Zhang

**Affiliations:** 1 Department of Diagnostic Ultrasound and Echocardiography, Sir Run Run Shaw Hospital, Zhejiang University College of Medicine, Technical Guidance Center for Fetal Echocardiography of Zhejiang Province and Sir Run Run Shaw Institute of Clinical Medicine of Zhejiang University, Hangzhou, China; 2 Department of Ultrasound, Hangzhou Women’s Hospital, Hangzhou, China; 3 Prenatal Diagnostic Center, Lishui Maternity and Child Health Care Hospital, Lishui, China

**Keywords:** Fetal heart quantification, cardiac function, prenatal prediction, coarctation of the aorta (CoA), fetal heart analysis

## Abstract

Coarctation of the aorta (CoA) ranks among the most prevalent congenital heart defects and poses a life-threatening risk if left undiagnosed. Herein, we utilized fetal heart quantification (HQ) technology to improve the prenatal prediction of CoA. A retrospective analysis was conducted on 64 fetal cases with suspected aortic arch constriction, identified through prenatal ultrasound findings between November 2020 and March 2022 at the Department of Ultrasound, Sir Run Run Shaw Hospital, Zhejiang University. According to the follow-up results, these cases were divided into two groups: 35 cases confirmed as CoA by postpartum surgery or induction, and 29 cases initially suspected of CoA prenatally but subsequently ruled out postnatally. Additionally, 88 cases of normal fetuses were randomly selected as the control group. Both conventional M-mode ultrasound techniques and Fetal HQ software were utilized for fetal analysis across all groups. Parameters related to the heart were measured, including fetal 4-CV length, width, global spherical index (GSI), mitral annular plane systolic excursion (MAPSE), areas and ratios of the left and right ventricles, as well as lengths and ratios of the left and right ventricles. Functional measurements of the left and right ventricles included ejection fraction (EF), fractional area change (FAC), global longitudinal strain (GLS), fractional shortening (FS), end-diastolic diameter (ED), and sphericity index (SI). Left ventricular (LV)-GLS, LV-FAC, LV-EF, and LV-EF Z-score could potentially differentiate between true CoA and false CoA or normal groups and serve as potential indicators for the clinical diagnosis of CoA. The receiver operating characteristic (ROC) curves indicated that LV-GLS and LV-EF Z-score have the greatest predictive power for CoA diagnosis. The segments 6–12 of FS in the confirmed CoA group were significantly lower than those in the false CoA and normal groups. Fetal HQ technology, by assessing changes in the size and shape of the heart, can provide relatively reliable parameter support for the prenatal diagnosis of fetal aortic coarctation.

## Introduction

Coarctation of the aorta (CoA) refers to a congenital narrowing of the descending part of the aortic arch, typically occurring around the distal left subclavian artery and the site of the ductus arteriosus insertion [1]. It is a duct-dependent congenital heart disease, accounting for approximately 4%–6% of congenital heart diseases [2–4]. Although the evaluation of the aortic arch has become an important part of fetal cardiac ultrasound examination, the sensitivity of prenatal ultrasound diagnosis of CoA is low [3, 5]. A survey on congenital heart disease by Lytzen et al. [3] reported that the prenatal detection rate of major fetal cardiac malformations increased from 4.50% in 1996 to 71.00% in 2013, but the detection rate of fetal CoA remained low (only 21.70%).

In addition, the results of a systematic review and meta-analysis of 12 studies showed that the false-positive rate of fetal CoA diagnosis using the length and area of the left and right ventricular end-diastole ranged from 0% to 67% [6]. Since normal fetal aortic isthmus blood flow accounts for only 10%–15% of the combined cardiac output [7], the blood flow in the isthmus is relatively poor compared with other segments of the aorta. Therefore, under normal circumstances, the isthmus diameter may be mildly narrowed [7]. Any factor that affects left ventricular (LV) output can affect the isthmus diameter, so such fetuses may also show ultrasound images similar to CoA. The significance of prenatal CoA diagnosis has been underscored by its ability to enhance perioperative outcomes and neonatal survival. This is achieved through planned delivery in specialized tertiary care centers and early initiation of prostaglandin treatment to forestall duct closure.

Various echocardiographic metrics have been proposed to augment the prenatal detection rate of CoA. These include two-dimensional measurements of ventricular inflow and outflow tracts, Z-scores derived from these measurements, comparison of diameters between left and right structures, Doppler signals, and additional features like the presence of a persistent left superior vena cava (LSVC), visualization of a juxtaductal shelf, and the carotid-subclavian index [6, 8–19].

The fetal heart quantification (Fetal HQ) technique utilizes two-dimensional speckle tracking technology to separately track the endocardium of the left and right ventricles of the fetus, dividing them into 24 equally spaced segments. This allows for quantitative analysis of the overall size, shape, and contractile function of the fetal heart and its left and right ventricles. Briefly, the acquired dynamic four-chamber heart images (in 4-D format) are imported into the Fetal HQ software package for analysis [20]. The end-diastole (ED) is determined, and measurements of the long axis (L), transverse width (TW), and area (A) of the four-chamber heart at ED are taken. The software automatically calculates the heart area and the global sphericity index (GSI). The technique also provides the areas (ED-A) and long axes (ED-L) of the left and right ventricles, as well as the fractional area change (FAC), global longitudinal strain (GLS), LV rejection fraction (LVEF), 24-segment sphericity index (SI), fractional shortening (FS), and their Z-scores [20–22].

In this study, Fetal HQ technology was utilized to quantitatively analyze the size, shape, and contractile function of the hearts of 35 fetuses with CoA confirmed by postpartum surgery or induction, as well as 29 cases that were suspected of CoA prenatally but ultimately found to be negative postnatally. Additionally, 88 cases of normal fetuses were included as healthy controls. The primary objective of this study was to retrospectively evaluate institutional practices concerning prenatal CoA diagnosis and the subsequent perinatal and postnatal management of fetuses with suspected CoA. Furthermore, we aimed to validate the effectiveness of previously proposed echocardiographic predictors of postnatal CoA, especially in cases where fetuses were referred to a tertiary care center from external facilities during late gestation.

## Materials and methods

### Study subjects

A retrospective review was conducted on 68 cases of prenatal ultrasound suggesting fetal aortic arch coarctation at Sir Run Run Shaw Hospital from Month 2020 to March 2022. Among them, one case was diagnosed postnatally as an interrupted aortic arch (IAA), three cases were lost to follow-up, and the actual pathological inclusion was 65 cases. Based on the follow-up results, the cases were divided into two groups: confirmed by postnatal surgery (*n* ═ 35) or termination of pregnancy (*n* ═ 30). Fetuses identified as CoA after delivery or induced labor by surgery were included in the CoA group (35 cases). Fetuses without CoA identified by postpartum cardiac ultrasound were included in the false CoA group (29 cases). A total of 88 normal fetal cases were randomly selected as the normal control group. Inclusion criteria: (1) singleton pregnancies; (2) physically healthy pregnant women; (3) without high-risk factors; (4) the ultrasound examination in the normal group did not reveal fetal cardiac structural or functional abnormalities, nor were there any other anomalies; (5) all pregnant women underwent quantitative analysis using Fetal HQ technology; (6) All pregnant women were informed of the accuracy and limitations of fetal echocardiography before the examination and signed an informed consent form for fetal cardiac ultrasound examination (CON157 10/13). Exclusion Criteria: (1) Prenatal diagnosis suggesting fetal genetic abnormality; (2) Hypoplastic left heart syndrome; (3) Transposition of the great arteries; (4) Double-outlet right ventricle; (5) Illegible image; (6) Lack of postnatal follow-up data. This study was approved by the Ethics Committee of the Affiliated Sir Run Run Shaw Hospital of Zhejiang University School of Medicine.

### Instrument

GE Voluson E10 color Doppler ultrasound with Fetal HQ software, probe models C6-1 and C9-2, frequencies ranging from 1 to 6 and 2 to 9 MHz, respectively, equipped with the Fetal HQ software package.

### Methods

Instruct the pregnant woman to lie on her side and conduct routine measurements to determine fetal size and gestational age. The age of the pregnant women was recorded, followed by measuring the mitral annulus displacement (MAD) of each fetus. Mitral annular plane systolic excursion (MAPSE) was calculated from the mean of the lateral and medial annular excursions. Perform a routine fetal echocardiography examination, taking the apical four-chamber view, and try to position the apex toward the probe to minimize interference factors such as fetal movement, ensuring clear visualization of the ventricular cavity and endocardial surface with a frame rate >80 Hz, saving a 2–3 s dynamic image of the apical four-chamber view. Then measure the area, length, width, and GSI of the fetal 4-CV at ED. Enter the Fetal HQ system, combine M-mode ultrasound with two-dimensional dynamic images to determine the end-diastolic and end-systolic time nodes based on the opening and closing states of the valves, place the sampling points at the junctions of the mitral valve, tricuspid valve, and ventricle, and at the apex of the left and right ventricles, and automatically trace the endocardial speckle tracking trajectory. The Fetal HQ software package divides the left and right ventricles into 24 segments, obtains dynamic tracking curves of the ventricular endocardium, and calculates the LV ejection fraction (EF), LV fraction area change (LV-FAC), right ventricular fraction area change (RV-FAC), LV-GLS, right ventricular (RV)-GLS, 24-segment short-axis shortening rate FS, LVED area, RVED area, SI, and their Z-scores (EF Z-score and LV-FAC Z-score).

**Figure 1.**
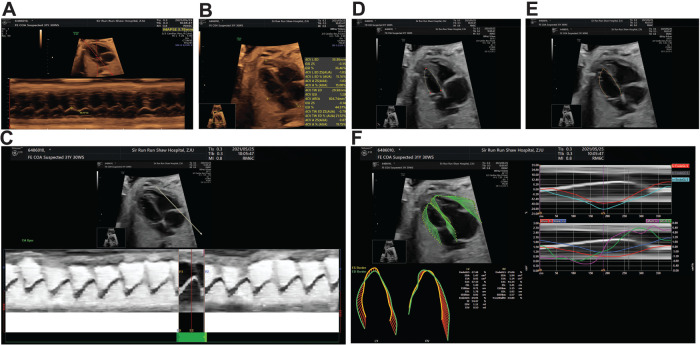
**Data acquisition flowchart.** (A) An M-shaped curve was used to measure mitral ring systolic displacement (MAPSE); (B) The long diameter and transverse diameter of the heart were measured at the end of diastole to obtain the area and global sphericity index (GSI); (C) End-diastolic systolic nodes were determined in M mode; (D) The endocardial surface was traced at the end of ventricular systole; (E) The endocardial surface of the end-diastolic ventricle was depicted; (F) Fetal HQ automatically calculates all parameters and displays the results. The long diameter of the heart is indicated by the red arrow; the transverse diameter of the heart is indicated by the yellow arrow; the blue arrow indicates the tracing along the endocardial border during left ventricular systole; the green arrow indicates the tracing along the endocardial border during left ventricular diastole. Fetal HQ: Fetal heart quantification.

### Reproducibility test

Ten cases were randomly selected from the normal group, and three or four cases were randomly selected from the CoA group and the false CoA group, respectively. Physicians with the same qualifications measured the data independently using the Fetal HQ software to conduct inter-rater reproducibility tests. Two weeks later, the same physicians re-measured the data using the Fetal HQ software for intra-rater reproducibility tests on the randomly selected 17 fetal cases from these three groups.

### Ethical statement

This study was approved by the ethical committee of Sir Run Run Shaw Hospital, Zhejiang University College of Medicine, with the approval number EC-SRRSH-ZJU-2020-3301-0002.

### Statistical analysis

Comparisons were made among the confirmed CoA fetuses (*n* ═ 35), false CoA fetuses (*n* ═ 29), and 88 cases in the control group. The Statistical Package for the Social Sciences (SPSS, version 26.0, SPSS Inc., Chicago, IL, USA) was used for data analysis. The Kolmogorov–Smirnov test was used to test the normality of the distribution. For normally distributed or approximately normally distributed continuous data, the mean ± SD was used for representation, and a one-way analysis of variance was used for comparisons among the three groups. For skewed distribution continuous data, the median (1st quartile, 3rd quartile) was used for representation, and the non-parametric Kruskal–Wallis test was applied. The diagnostic efficacy of single and combined indicators was analyzed using the receiver operating characteristic (ROC) curve, and the predictive power of different indicators was evaluated using the Area Under the ROC curve (AUC) of the ROC curve.

## Results

### The studied population was matched for maternal and gestational age at the time of the ultrasound examination

**Figure 2.**
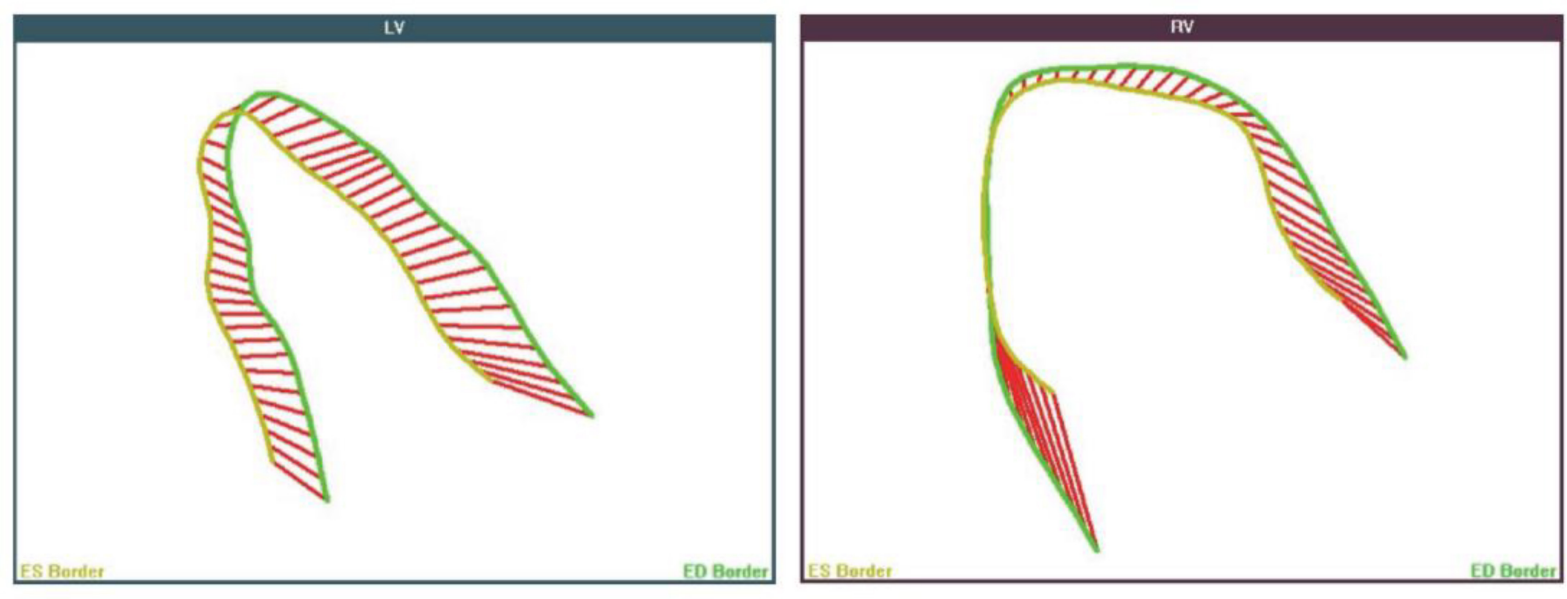
**Representative images of Fetal HQ from fetus in CoA group.** Left: Left ventricle; Right: Right ventricle; CoA: Coarctation of the aorta; Fetal HQ: Fetal heart quantification.

**Figure 3.**
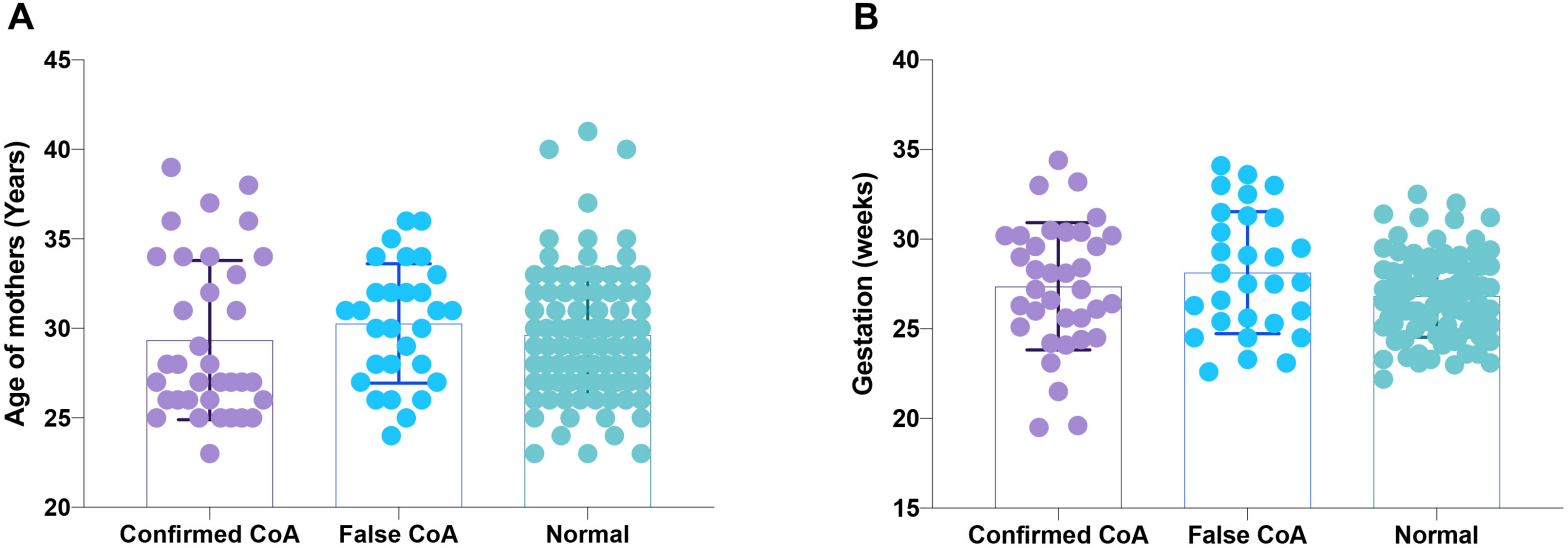
**Prenatal details of the study population (Confirmed CoA: fetuses with suspected CoA and confirmed postnatally; False CoA: fetuses with suspected CoA but found normal postnatally; Normal: fetuses without suspected CoA).** (A) Age of mothers at the time of the ultrasound examination; (B) Gestational age of fetuses at the time of the ultrasound examination. CoA: Coarctation of the aorta.

The data acquisition flowchart is shown in Figure 1. Representative images of Fetal HQ from CoA fetuses are shown in Figure 2. The confirmed CoA group, false CoA group, and normal group were matched for the age of mothers at the time of the ultrasound examination and the gestational age of fetuses at the time of the ultrasound examination (Figure 2). The average maternal age in the confirmed CoA group was 27 years, with an average gestational age of 27.36 weeks at the time of the ultrasound examination. In the false CoA group, the average maternal age was 31 years, with an average gestational age of 28.13 weeks at the time of the ultrasound examination. Among the randomly selected 88 normal fetuses, the average maternal age was 29 years, with an average gestational age of 26.82 weeks at the time of the ultrasound examination (Figure 3 and Table 1).

**Table 1:** Prenatal ultrasound findings of the study population (Confirmed CoA: fetuses with suspected CoA and confirmed postnatally; False CoA: fetuses with suspected CoA but found normal postnatally; Normal: fetuses without suspected CoA)

**Groups**	**G1**	**G2**	**G3**	***P* value**		
	**Confirmed CoA (*n* ═ 35)**	**False CoA (*n* ═ 29)**	**Normal (*n* ═ 88)**	***P*1 (G1 vs G2)**	***P*2 (G1 vs G3)**	***P*3(G2 vs G3)**
Age of mother (year)	27 (26, 34)	31 (27.5, 32.5)	29 (27, 32)	n.s.	n.s.	n.s.
Gestation (week)	27.36 ± 3.54	28.13 ± 3.40	26.82 ± 2.29	n.s.	n.s.	n.s.
MAPSE (mm)	6.45 (4.67, 6.85)	6.04 (4.48, 6.51)	5.15 (4.02, 5.78)	n.s.	**<0.001**	**<0.001**
GSI	1.16 ± 0.06	1.18 ± 0.06	1.23 ± 0.06	n.s.	**<0.001**	**<0.001**
LVED area (mm^2^)	1.16 ± 0.453	1.23 ± 0.39	1.38 ± 0.36	n.s.	**0.007**	**0.015**
RVED area (mm^2^)	1.65 (0.98, 1.87)	1.69 (1.03, 2.41)	1.51 (1.05, 1.89)	n.s.	n.s.	n.s.
LVED/RVED ratio	1.09 (0.74, 1.49)	0.99 (0.77, 1.10)	1.22 (0.93, 1.33)	n.s.	**0.025**	**<0.001**
LV Length (mm)	1.66 ± 0.33	1.70 ± 0.34	1.82 ± 0.25	n.s.	n.s.	**0.013**
RV Length (mm)	1.64 (1.22, 1.74)	1.69 (1.33, 2.18)	1.78 (1.35, 1.97)	n.s.	**0.014**	**0.012**
LV Length/RV Length ratio	1.34 (1.01, 1.50)	1.27 (1.01, 1.37)	1.22 (1.06, 1.34)	n.s.	n.s.	n.s.
LV-GLS (%)	22.24 (15.27, 25.75)	24.39 (19.26, 28.62)	26.45 (19.49, 30.06)	**0.019**	**<0.001**	n.s.
RV-GLS (%)	24.19 (16.55, 27.53)	23.77 (19.04, 26.82	26.17 (19.63, 28.59)	n.s.	**0.034**	**0.027**
LV-FAC (%)	39.37 ± 7.02	43.96 ± 7.07	46.89 ± 6.32	**0.006**	**<0.001**	**0.041**
RV-FAC (%)	40.04 ± 8.13	42.26 ± 5.38	42.17 ± 5.98	n.s.	n.s.	n.s.
LV-EF (%)	54.66 ± 7.98	59.55 ± 9.32	62.41 ± 8.48	**0.015**	**<0.001**	**<0.001**

CoA: Coarctation of the aorta; MAPSE: Mitral annular plane systolic excursion; GSI: Global spherical index; LV: Left ventricular; RV: Right ventricular; LV-FAC: Left ventricular fraction area change; RV-FAC: Right ventricular fraction area change; LV-ED: Left ventricular end-diastole; RV-ED: Right ventricular end-diastole; GLS: Global longitudinal strain; LV-EF: LV rejection fraction.

### Analyzing the size and morphology of fetal hearts using Fetal HQ does not accurately diagnose CoA from false CoA

**Figure 4.**
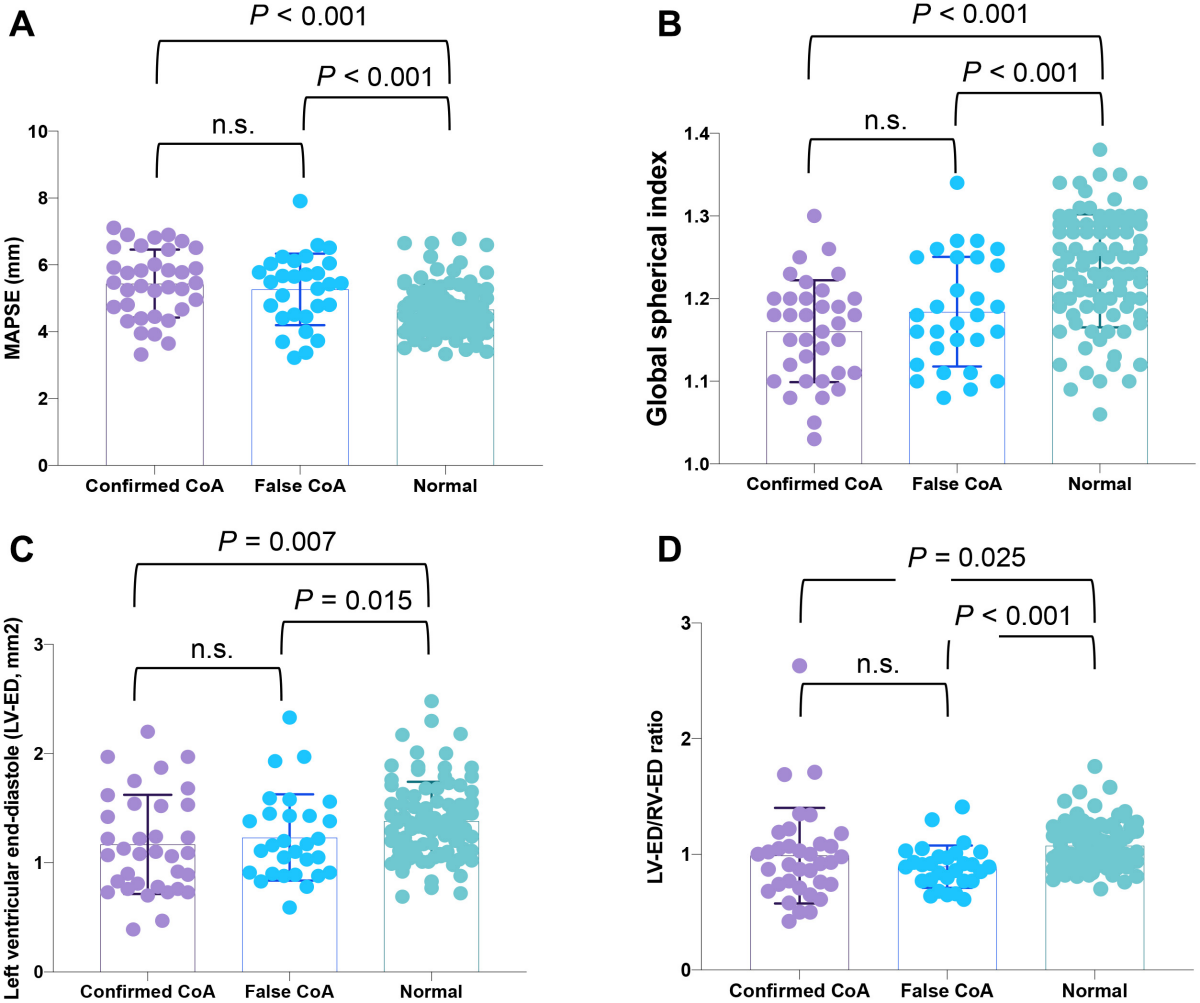
**Fetal heart descriptive parameters obtained through the Fetal HQ technique do not distinguish CoA fetuses from false-positive cases.** (A) Mitral annular plane systolic excursion (MAPSE) measurements in confirmed CoA, false CoA and normal groups; (B) Global sphericity index in confirmed CoA, false CoA, and normal groups; (C) Left ventricular end-diastole (LV-ED) area measured in confirmed CoA, false CoA, and normal groups; (D) Left ventricular end-diastole area to right ventricular end-diastole (RV-ED) area ratio in confirmed CoA, false CoA, and normal groups. CoA: Coarctation of the aorta; Fetal HQ: Fetal heart quantification.

To verify whether Fetal HQ can differentiate between CoA, false CoA, and normal fetal groups, an analysis comparing the size and morphological changes of fetal hearts was conducted. The comparative analysis revealed no statistical difference in the RVED area among the three study groups (*P* > 0.05). Similarly, there was no statistical difference in the LV length to RV length ratio among the three groups (*P* > 0.05), indicating that RVED area and LV length/RV length ratio are not effective in differentiating between CoA fetuses and normal fetuses. Further comparison of GSI, LVED area, LVED/RVED area ratio, and RV length revealed significant statistical differences between the true CoA/false CoA groups and normal fetuses (all *P* < 0.05). However, these indicators failed to distinguish between the true CoA and false CoA groups, indicating a risk of false-positive results when diagnosing CoA using these indicators. Additionally, we observed a significant difference in LV length between the true CoA and normal fetal groups, but no difference between the false CoA and normal fetal groups (Table 1 and Figure 4). These findings suggest that analyzing and comparing the size and morphology of fetal hearts may not provide an accurate CoA diagnosis.

### LV-GLS, LV-FAC, and EF potentially differentiate CoA from false CoA and normal fetuses

**Figure 5.**
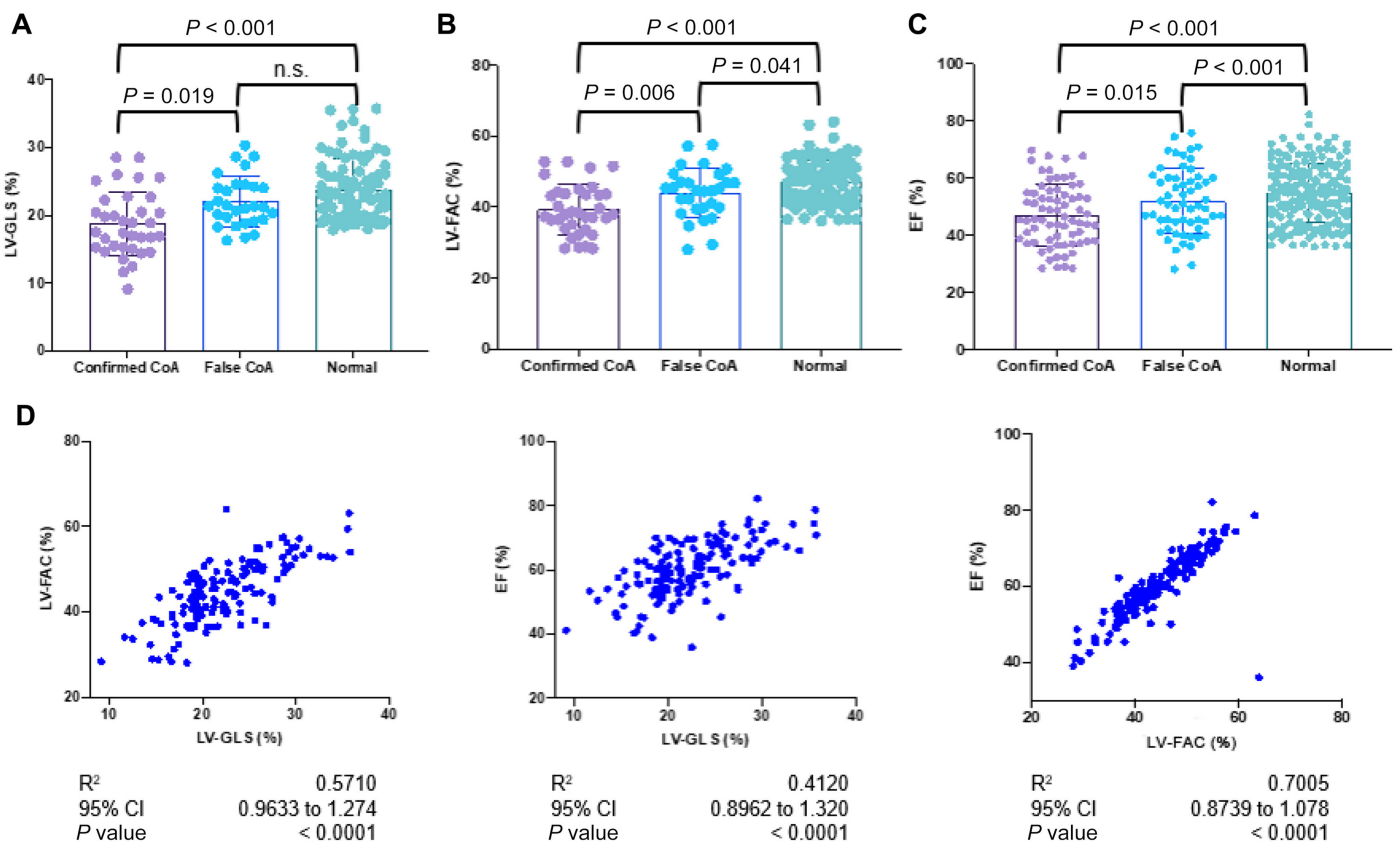
**Fetal heart functional assessment via Fetal HQ technique differentiates CoA fetuses from false-positive cases.** (A) LV-GLS measurements in confirmed CoA, false CoA, and normal groups; (B) LV-FAC in confirmed CoA, false CoA, and normal groups; (C) EF measured in confirmed CoA, false CoA, and normal groups; (D) The correlation among LV-FAC, LV-GLS, and EF. CoA: Coarctation of the aorta; EF: Ejection fraction; LV-FAC: Left ventricular fractional area change; LV-GLS: Left ventricular global longitudinal strain.

The progression of CoA significantly affects fetal cardiac function; therefore, this study further measured fetal cardiac functional parameters. There was no statistical difference in RV-FAC among the three groups of study cases (*P* > 0.05), while MAPSE and RV-GLS could not differentiate between the true CoA and false CoA groups. Notably, statistical differences were observed in LV-FAC and EF among the three groups (*P* < 0.05). Furthermore, LV-GLS could significantly differentiate between the true CoA and false CoA groups, with no difference between the false CoA and normal groups (Table 1). In the confirmed CoA group, the LV-GLS was 22.24 (15.27, 25.75), and in the false CoA group or normal fetal group, it was 24.39 (19.26, 28.62), and in the normal fetal group, it was 26.45 (19.49, 30.06) (Figure 5A). LV-FAC in the true CoA group, false CoA group, or normal fetal group was 39.37 ± 7.02, 43.96 ± 7.07, and 46.89 ± 6.32, respectively (Figure 5B). LV-EF in the true CoA group, false CoA group, or normal fetal group was 54.66 ± 7.98, 59.55 ± 9.32, and 62.41 ± 8.48, respectively (Figure 5C). LV-GLS, LV-FAC, and EF were significantly lower in the CoA group compared to the false CoA group or normal fetal group. Correlation analysis among LV-GLS, LV-FAC, and EF showed a certain correlation among these three indicators, with the strongest correlation between LV-GLS and LV-EF, *R*^2^ ═ 0.7 (Figure 5D). In conclusion, through the analysis of fetal cardiac size, morphology, and functional parameters, it is suggested that LV-GLS, LV-FAC, and EF could potentially differentiate between true CoA and false CoA or normal groups and serve as potential indicators for the clinical diagnosis of CoA.

### Z-scores of EF and LV-FAC distinguish false CoA from CoA fetuses

**Figure 6.**
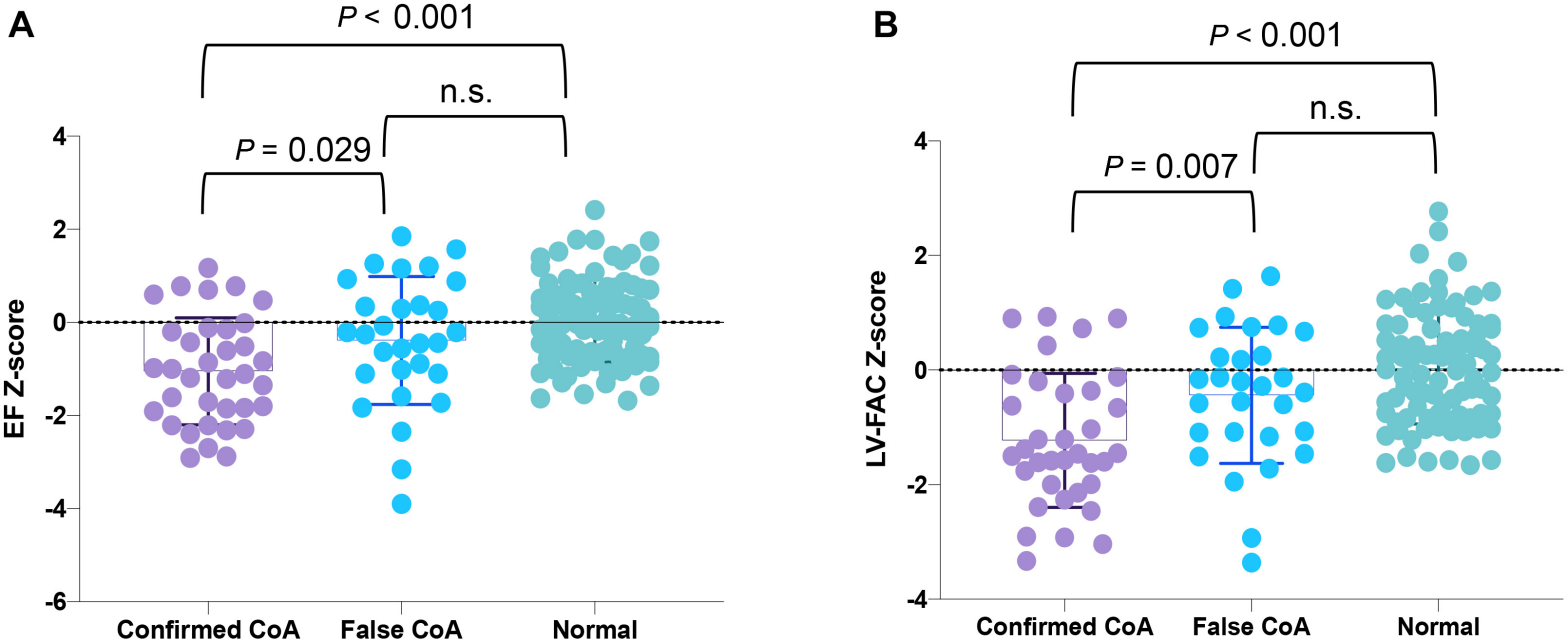
**The Z-scores differentiate CoA fetuses from false-positive cases.** (A) Z-score of EF calculated in confirmed CoA, false CoA, and normal groups; (B) Z-score of LV-FAC calculated in confirmed CoA, false CoA, and normal groups. CoA: Coarctation of the aorta; EF: Ejection fraction; LV-FAC: Left ventricular fractional area change.

To further validate the impact of different indicators on the diagnosis of CoA, we analyzed the Z-scores of EF and LV-FAC. The average Z-score of LV-EF in the CoA group (−1.05) was significantly lower than that in the false CoA group (−0.39) and the normal group (0.03). Furthermore, in the Z-score of LV-FAC, the average Z-score of LV-EF in the CoA group (−1.23) was significantly lower than that in the false CoA group (−0.44) and the normal group (0.03) (Figure 6).

**Figure 7.**
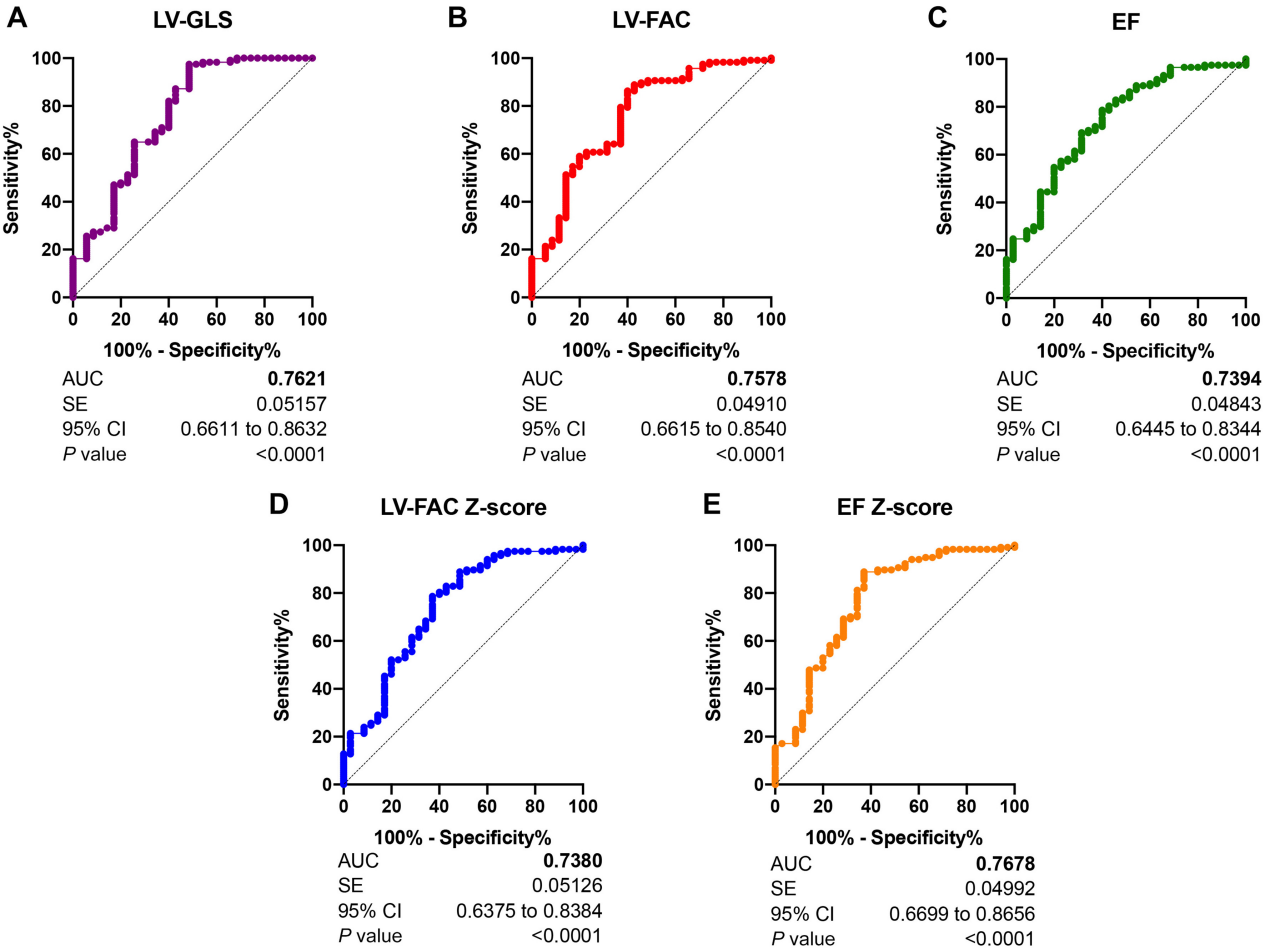
**The ROC curves of parameters that differentiate CoA fetuses from false-positive cases, including: (A) LV-GLS; (B) LV-FAC; (C) EF; (D) The Z-score of LV-FAC; and (E) The Z-score of EF.** Patients were defined as in the confirmed CoA group, while normal controls were defined as in the false CoA and normal groups. CoA: Coarctation of the aorta; EF: Ejection fraction; ROC: Receiver operating characteristic; LV-FAC: Left ventricular fractional area change; LV-GLS: Left ventricular global longitudinal strain.

To assess the power of these indicators in CoA diagnosis, we evaluated the predictive power of different indicators using the area under the curve (AUC) of the ROC curve. We defined the CoA group as the positive group, and the false CoA group and the normal group were defined as the negative group. The AUC values were as follows: LV-EF Z-score (0.7678) > LV-GLS (0.7621) > LV-FAC (0.7578) > LV-EF (0.7394) > LV-FAC Z-score (0.7380). This indicates that LV-EF Z-score and LV-GLS have the greatest predictive power for CoA (Figure 7).

**Figure 8.**
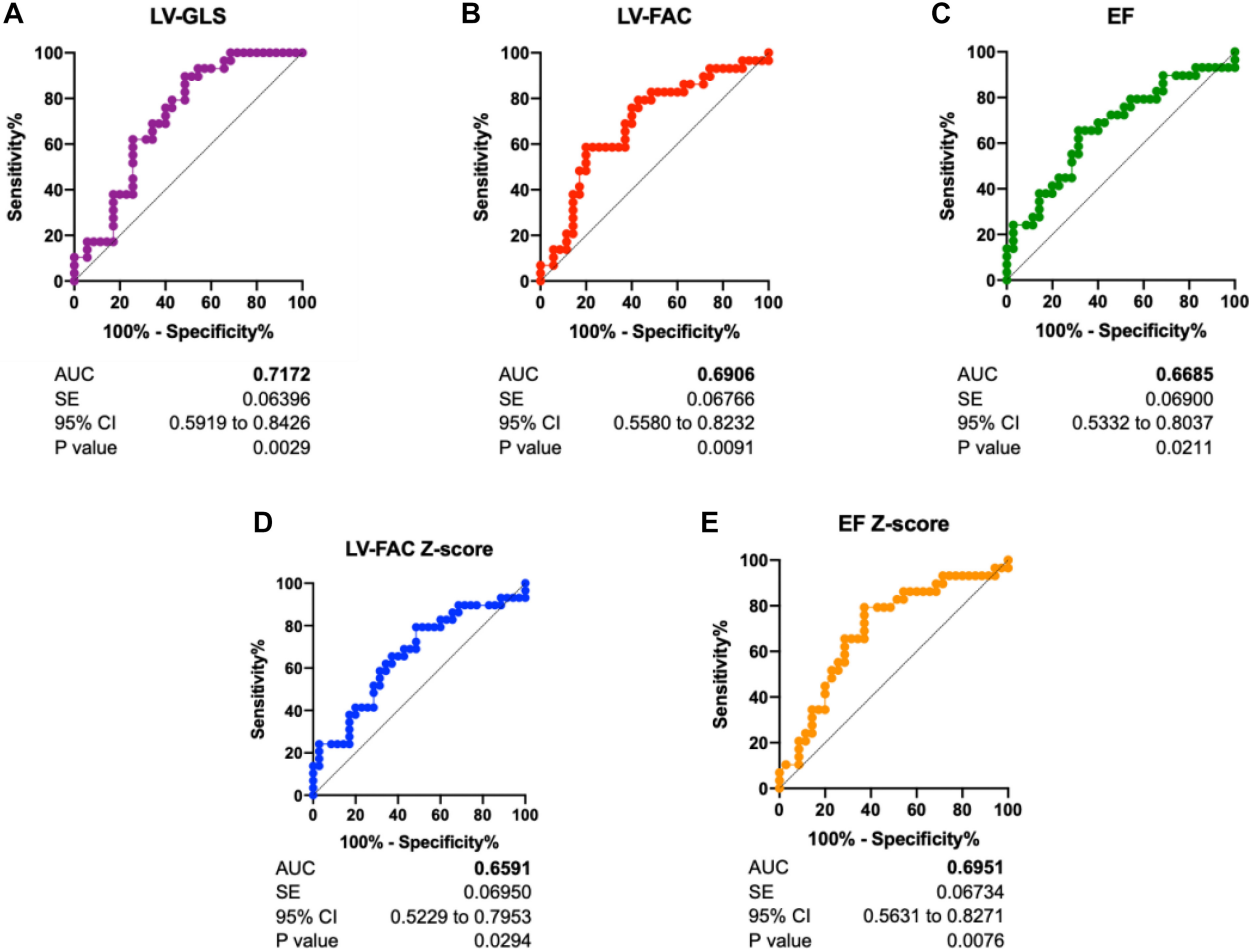
**The ROC curves of parameters that differentiate CoA fetuses from false-positive cases, including: (A) LV-GLS; (B) LV-FAC; (C) EF; (D) The Z-score of LV-FAC; and (E) the Z-score of EF.** Patients were defined as in the confirmed CoA group, while controls were defined as in the false CoA group. CoA: Coarctation of the aorta; EF: Ejection fraction; ROC: Receiver operating characteristic; LV-FAC: Left ventricular fractional area change; LV-GLS: Left ventricular global longitudinal strain.

False positives in CoA diagnosis are currently a problem encountered in the diagnostic process. Therefore, to better distinguish between true CoA and false CoA, we again defined the CoA group as the positive group and the false CoA group as the negative group, enabling a clearer distinction between true and false CoA. The results showed that the AUC of the ROC curve was as follows: LV-GLS (0.7172) > LV-EF Z-score (0.6951) > LV-FAC (0.6906) > LV-EF (0.6685) > LV-FAC Z-score (0.6591). This indicates that LV-GLS and LV-EF Z-score have the greatest predictive power for CoA (Figure 8).

**Figure 9.**
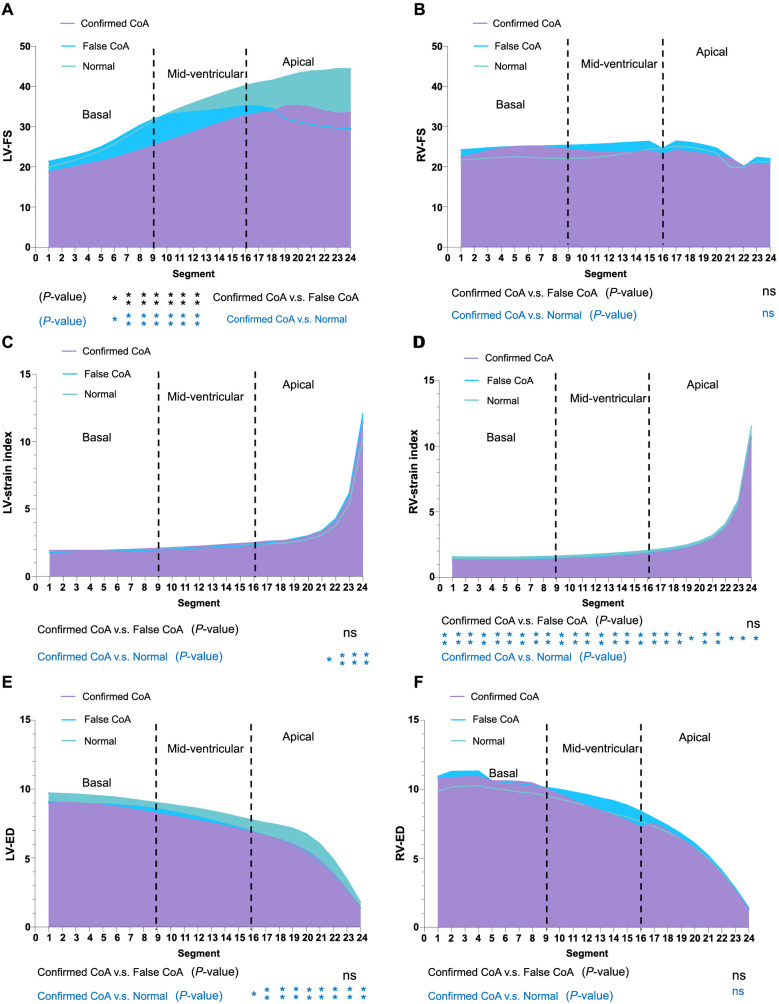
**The FS, strain index, and ED of the 24 segments of the fetal left and right ventricles in confirmed CoA, false CoA, and normal groups.** (A) FS of the 24 segments of the fetal left ventricle; (B) FS of the 24 segments of the fetal right ventricle; (C) Strain index of the 24 segments of the fetal left ventricle; (D) Strain index of the 24 segments of the fetal right ventricle; (E) ED of the 24 segments of the fetal left ventricle; (F) ED of the 24 segments of the fetal right ventricle. *P < 0.05; **P < 0.01. CoA: Coarctation of the aorta; FS: Fractional shortening; ED: End-diastolic dimension; LV: Left ventricular; RV: Right ventricular.

To further investigate the differences among confirmed CoA, false CoA, and normal groups, we measured the FS, strain index, and end-diastolic dimension (ED) of the 24 segments of the fetal left ventricle in these groups. As shown in Figure 9, when comparing the confirmed CoA group with the false CoA and normal groups, the LV-FS of segments 6–12 in the CoA group was significantly lower compared to the control group (false CoA and normal groups) (*P* < 0.05, Figure 9A). There was no statistically significant difference in the RV-FS and RV-ED of the 24 segments between the CoA group and the false CoA/normal groups (*P* > 0.05, Figure 9B and 9F). Although the apical segments of LV-strain index (Figure 9C) and LV-ED (Figure 9E) and all segments of RV-strain index (Figure 9D) could distinguish the normal group from the CoA group, they do not differentiate between CoA and false CoA. Therefore, these parameters are not promising for clinical diagnosis.

## Discussion

CoA is one of the most prevalent congenital heart defects and poses a significant life-threatening risk if undiagnosed. However, despite advancements in prenatal screening for congenital heart defects and the use of sophisticated techniques that combine measurements of cardiac structures with Doppler analysis during fetal echocardiographic assessments, the sensitivity of prenatal ultrasound diagnosis for CoA remains low, with a high false-positive rate. Therefore, diagnosing CoA in utero continues to be challenging [19].

Prenatal ultrasound typically predicts the degree of CoA stenosis using the ratio of the right ventricular to LV transverse diameter [23]. However, ventricular size is affected by the internal diameter of the foramen ovale and atrioventricular orifice blood flow, which may not fully reflect aortic arch development [23]. In addition, the study by Gomez-Montes et al. [14] predicted aortic arch development using the ratio of the pulmonary artery to aortic diameter. Unfortunately, CoA is often accompanied by aortic dysplasia, and CoA cannot be accurately judged using the ratio of pulmonary artery to aortic diameter alone [14]. A review of cases from January 2020 to March 2022 identified 68 prenatal ultrasound indications of fetal aortic arch narrowing at the hospital, but only 35 cases were confirmed by postnatal surgery or terminated pregnancies. This confirms that the specificity of prenatal CoA diagnosis remains poor, with a high false-positive rate. In the present study, we utilized Fetal HQ technology and carefully measured fetal heart size and morphological changes. Interestingly, the comparison of GSI, LVED area, LVED/RVED area ratio, and RV length revealed significant statistical differences between true CoA and false CoA groups when compared to normal fetuses (*P* < 0.05), suggesting that analyzing and comparing the size and morphology of fetal hearts may not accurately diagnose CoA. Due to the progressive nature of fetal CoA, its morphological and functional changes do not always match. Additionally, Sivanandam et al. [24] noted that some fetuses with suspected CoA received a postpartum diagnosis of non-CoA along with an imbalance in the right/LV ratio. Therefore, fetal echocardiographic diagnosis of CoA requires a comprehensive analysis of multiple indicators rather than relying solely on morphological parameters.

The reproducibility of Fetal HQ in fetal heart analysis has been demonstrated [25]. This technology divides the ventricles into 24-segment quantitative assessments, providing a closer approximation to the original ventricular morphology and overcoming the limitations of two-dimensional single-section measurements [25]. Upon analysis of fetal cardiac functional parameters, we found that there was no statistical difference in RV-FAC among the three groups of study cases (*P* > 0.05), while MAPSE and RV-GLS could not differentiate between true CoA and false CoA groups. Interestingly, there were statistical differences in LV-FAC and EF among the three groups (*P* < 0.05). Furthermore, LV-GLS could significantly differentiate between true CoA and false CoA groups, with no difference between false CoA and normal groups. In conclusion, through the analysis of fetal cardiac size, morphology, and functional parameters, it is suggested that LV-GLS, LV-FAC, and LV-EF could potentially differentiate between true CoA and false CoA and serve as potential indicators for clinical diagnosis of CoA. Furthermore, DeVore et al. [26] compared neonates with CoA to healthy neonates and found that there were also significant differences between the two groups in the apical four-chamber view as well as in the shape and size of the left and right ventricles, longitudinal and transverse systolic function, cardiac output, and EF. Interestingly, the data strongly suggested that LV parameters are more pronounced than those in the right ventricle. These differences are most likely associated with clinical observations. In isolated CoA, the four-chamber view always shows a decrease in the size of the left ventricle and a relative increase in the size of the right ventricle.

Statistical analysis identified that the Z-score of EF and Z-score of LV-FAC could also serve as sensitive parameters for distinguishing CoA from false-positive cases. These parameters struck the best balance between sensitivity and specificity for CoA diagnosis in fetuses with isolated cardiac asymmetry. Combining these parameters contributes to increasing prenatal ultrasound examination sensitivity, albeit with an expected reduction in specificity. Numerous echocardiographic parameters have been proposed to potentially enhance CoA detection rates and risk stratification. Previous studies revealed significant differences in several parameters among fetuses with CoA, particularly in left inflow and outflow tracts [26, 27]. When comparing the confirmed CoA patients to normal or false-positive CoA cases, the ROC curves suggested that LV-GLS and LV-EF Z-scores have the greatest predictive power for CoA. Overall, LV-GLS, LV-FAC, LVEF, EF Z-value, and LV-FAC Z-value can effectively diagnose the occurrence of CoA. This helps to further confirm the diagnosis in fetuses with suspected CoA as suggested by prenatal care and reduces the misdiagnosis of the fetus.

## Conclusion

In conclusion, the application of Fetal HQ technology in CoA diagnosis revealed that LV-GLS and LV-EF Z-score exhibit the greatest predictive power for CoA. However, there are still limitations in the study. Fetal HQ is analysis software reliant on the four-chamber view, which necessitates high-quality sections and frame rates. Its accuracy can be influenced by factors, such as maternal abdominal fat, fetal position, and fetal movement. Additionally, this study only analyzed the results of a single prenatal ultrasound examination for each case, without performing dynamic tracking and observation of changes in fetal cardiac parameters during pregnancy. The number of cases in this study is limited, and further validation with a larger sample size is necessary.

## Data Availability

Data generated in the present study are included in the manuscript or supplementary materials.
